# Optimizing Factors in Murine Whole-Organ Cochlea Culture

**DOI:** 10.3390/ijms26083908

**Published:** 2025-04-21

**Authors:** Andrea Tröger, Werner Bader, Timo Gottfried, Matthias Santer, Charles Schmit, Anneliese Schrott-Fischer, Joachim Schmutzhard

**Affiliations:** Department of Otorhinolaryngology, Medical University Innsbruck, 6020 Innsbruck, Austria

**Keywords:** cochlea organ culture, Bdnf, Ntf3, hypothermia, structure preservation, inner ear morphology

## Abstract

In 2008, Hahn et al. presented a method for cultivating a 3D organ culture of the cochlea. Although this method is well established, it is currently only applied to early postnatal animals. Given the known differences in regeneration and repair abilities between early postnatal and adult mammalian cochleae, our goal was to further develop and optimize this method to extend it beyond early postnatal animals to include adult mammalian cochleae. After rapidly dissecting the cochlea, it is opened and placed in a neurotrophin-containing culture medium. The culture is then maintained at 32 °C in a rotating bioreactor for 24 h. The combination of mild hypothermia (32 °C), quick cochlea dissection, and the addition of 10 ng/mL of Brain-derived neurotrophic factor (Bdnf) and 5 ng/mL of Neurotrophin 3 (Ntf3) to the culture medium ensures the complete cell survival of all cochlear cell types in 10-day-old mice. The modifications to the established method include the incorporation of neurotrophins (Bdnf and Ntf3) into the culture medium and cultivation under mild hypothermic conditions (32 °C). By introducing neurotrophins and cultivating at 32 °C, a 3D organ culture of the cochlea can also be established with 10-day-old mice. This in vitro model preserves all cochlear cell types under conditions similar to those found in vivo.

## 1. Introduction

Although many inner ear diseases are common and significantly impact daily life, their pathophysiology remains largely unknown. Neurobiological research on the inner ear is challenging due to its complex tissue structure and anatomy. To date, cell, tissue, and organ cultures have been used in inner ear research. However, cell and tissue cultures have the disadvantage of being detached from their physiological environment. In contrast, organ cultures preserve the entire organ and maintain its polarized fluid environment.

While organ cultures of the inner ear had been performed previously, 3D organ culture was a significant challenge until 2008, when Hahn et al. succeeded in using a rotating bioreactor system [1,2,3]. This method preserved the three-dimensional structure and unique fluid conditions of the cochlea by opening the bony labyrinth at the basal and apical turns, allowing the preservation of inner and outer hair cells, stereocilia bundles, and the integrity of the reticular lamina [3]. Maintaining the integrity of the stria vascularis (SV) is equally important for sustaining the physiological conditions of the cochlea, as the stria vascularis plays a critical role in generating the endocochlear potential through potassium circulation, which is essential for normal cochlea function and hearing [4]. This created an in vitro model of the cochlea with conditions close to those in vivo.

Due to the accessibility and availability of rodent cochleae, various animal models have been established. However, whole-organ cochlea culture has, so far, been limited to early postnatal animals [3,5,6]. Previous studies using cell cultures have shown differences in regeneration and repair between early postnatal and adult mammalian cochleae [7]. Therefore, research using whole-organ cochlea cultures should extend beyond early postnatal organs.

As noted, whole-organ cochlea cultures are challenging. Optimizing factors such as temperature, the speed of dissection, and the addition of neurotrophins may improve the process and enable the culturing of older animals. Currently, cochlea organ culture is performed at 37 °C, which represents body temperature. However, studies have shown that mild hypothermia can provide otoprotection by slowing metabolism, reducing oxidative stress, and involving cold-shock proteins [8]. These findings could enhance the structure and survival of cochlea organ cultures.

Preparation time is also crucial since inner ear hair cells remain viable for only a short period under hypoxic conditions [9]. Rapid cochlea dissection is essential to preserve hair cells in organ cultures. Additionally, modifying the culture medium, such as adding neurotrophins, could improve cochlear culture survival. Neurotrophins, such as Brain-derived neurotrophic factor (Bdnf) and Neurotrophin 3 (Ntf3), are essential in the inner ear. Their corresponding high-affinity receptors, TrkB and TrkC, are expressed early in development and overlap with the later expression domains of Bdnf and Ntf3, respectively. This receptor–ligand interaction is essential for the development and maintenance of spiral ganglion neurons (SGNs). A complete loss of both TrkB and TrkC results in the absence of SGNs [10,11,12,13]. However, their potential to improve culture conditions needs further investigation.

This study aimed to identify the factors that enhance whole cochlea culture conditions, with a focus on preparation time, culture temperature, and neurotrophic factor concentrations.

## 2. Results

**Figure 1:**
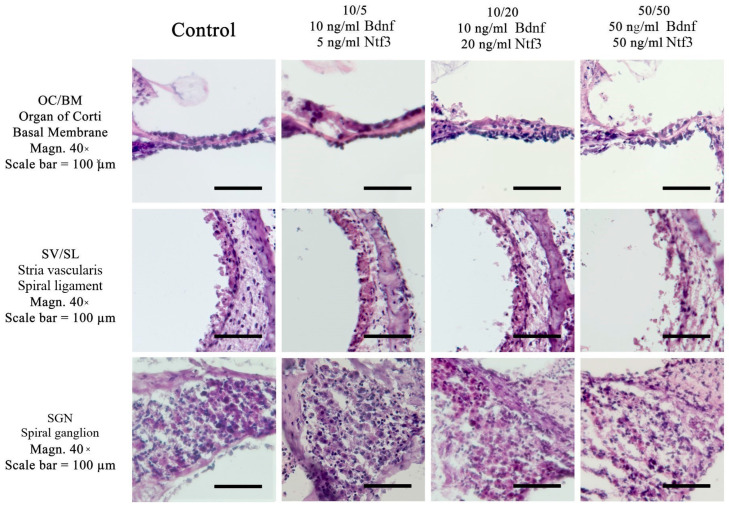
Comparison of different culture media with three different concentrations of neurotrophic factors at 37 °C in the slowly dissected cochlea. The control media is without a neurotrophin supplement. The concentrations used are grouped by column: 10/5 (10 ng/mL Brain-derived neurotrophic factor (Bdnf) and 5 ng/mL Neurotrophin 3 (Ntf3), 10/20 (10 ng/mL Bdnf and 20 ng/mL Ntf3), and 50/50 (50 ng/mL Bdnf and 50 ng/mL Ntf3). The lines in the images indicate the organ of Corti (OC), the stria vascularis (SV)/spiral ligament (SL), and the spiral ganglion neurons (SGNs). Experiments were performed at 37 °C in a rotary culture device. A standard Hematoxylin–eosin (HE) stain was used. Magnification = 40×; numerical aperture (NA) = 0.95; scale bar = 100 μm.

**Figure 2:**
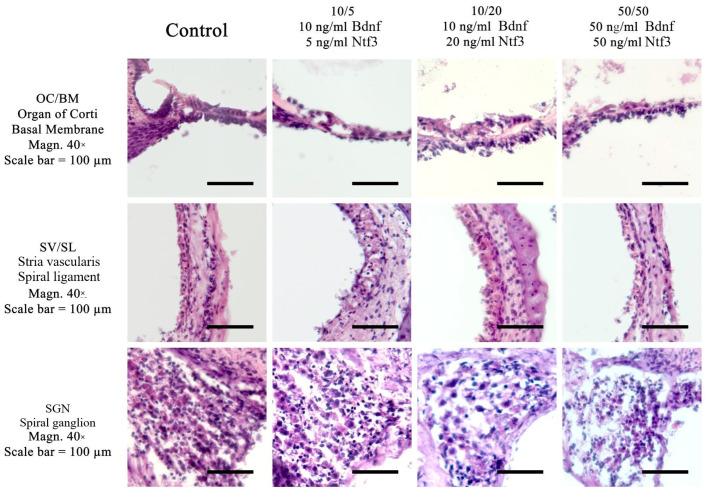
Comparison of different culture media with three different concentrations of neurotrophic factors at 32 °C in the quickly dissected cochlea, including a control medium without neurotrophins. The concentrations used were 10/5 (10 ng/mL Bdnf and 5 ng/mL Ntf3), 10/20 (10 ng/mL Bdnf and 20 ng/mL Ntf3), and 50/50 (50 ng/mL Bdnf and 50 ng/mL Ntf3). The experiments were performed at 32 °C in a rotary culture device. A standard HE stain was used. Magnification = 40×; NA = 0.95; scale bar = 100 μm.

The cell and structural preservation of the cochlea in various culture conditions were evaluated. Cultures were performed in either normothermic conditions (37 °C) or mild hypothermic conditions (32 °C). Furthermore, a comparison of different culture media with different concentrations of added Bdnf and Ntf3 was performed. Using hematoxylin and eosin (HE) stains, Figure 1 presents a comparison of the different culture media at 37 °C, while Figure 2 presents the comparison at 32 °C.

**Figure 3:**
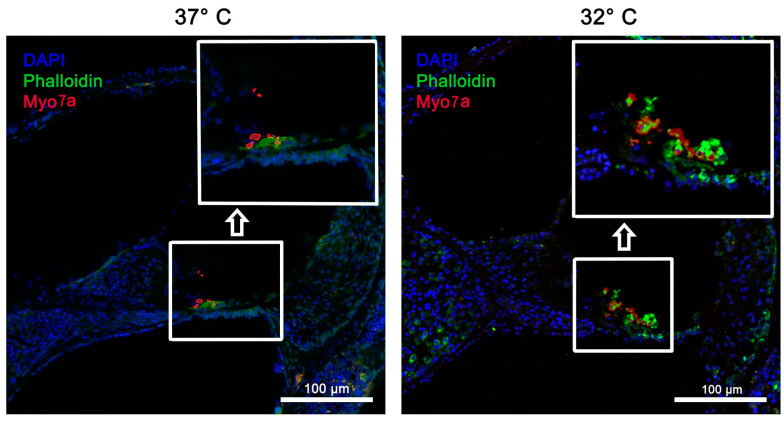
The structural preservation of inner ear tissue using the Myo7a marker at simulated physiological body temperature (37 °C) vs. simulated mild hypothermia (32 °C) in rotary culture, using a 10/5 concentration (10 ng/mL Bdnf and 5 ng/mL Ntf3) and the quickly dissected cochlea. Inner hair cells were well preserved and are indicated in red; blue = DAPI stain of cell nuclei; green = phalloidin (FITC) tissue fibers and connective tissue. Magnification = 40×; NA = 0.95; scale bar = 100 μm.

**Figure 4:**
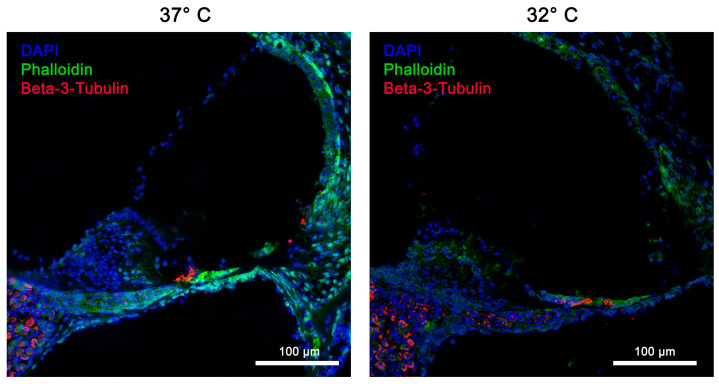
The structural preservation of inner ear tissue using the ß-III-tubulin marker at simulated physiological body temperature (37 °C) vs. simulated mild hypothermia (32 °C) in rotary culture using a 10/5 concentration (10 ng/mL Bdnf and 5 ng/mL Ntf3) and the quickly dissected cochlea. Nerve fibers, stained with ß-III-tubulin, are clearly visible and directly connected to the SGNs. All nerve fibers and ganglia cells are stained red; blue = DAPI stain of cell nuclei; green = phalloidin (FITC) tissue fibers and connective tissue. Inner hair cells (IHCs) and outer hair cells (OHCs) are well preserved and stained with phalloidin. Magnification = 40×; NA = 0.95; scale bar = 100 μm.

In addition to the HE stains, fluorescent immunohistochemistry was performed to further examine the integrity of the cochleae. Immunofluorescence staining was performed to visualize phalloidin, Myo7a, and ß-III-tubulin. In particular, the cell cultures performed at 32 °C showed increased fluorescence of phalloidin, indicating better preservation of the cytoskeleton’s organization in contrast to the culture at 37 °C. Myo7a and ß-III-tubulin could be detected at both 32 °C and 37 °C. However, intensified staining was seen at 32 °C, as seen in Figure 3 and Figure 4.

In addition, a quantitative evaluation of cell survival was performed, sorting the data by different areas of the cochlea under different culture conditions. The different areas were the spiral ganglion neurons (SGNs), organ of Corti (OC), and stria vascularis (SV)/spiral ligament (SL).

### 2.1. Spiral Ganglion Neurons

In the investigated area of the SGNs, the control group (37 °C, no neurotrophins) showed average cell maintenance of 170.57 ± 45.4 (standard deviation, SD) cells per investigated unit. Compared with the control group, both the addition of neurotrophins at the 10/5 concentration (10 ng/dL Bdnf and 5 ng/dL Ntf3) and the 10/20 concentration (10 ng/mL Bdnf and 20 ng/mL Ntf3) showed a statistically significant improvement in mean cell survival when combined with a quickly dissected cochlea and mild hypothermic culture conditions at 32 °C (*p*-values < 0.001 and 0.005). Using these modifications of the culture, a mean of 263.50 ± 15.94 (SD) cells per unit was preserved for the 10/5 concentration and 227.78 ± 18.57 (SD) cells per unit for the 10/20 concentration. The ß-III-tubulin immunofluorescence staining in Figure 4 shows significantly improved structural preservation of SGNs at 32 °C.

Even with slow treatment, adding 10 ng/mL Bdnf and 5 ng/mL Ntf3 or 10 ng/mL Bdnf and 20 ng/mL Ntf3 significantly improved cell maintenance at both 32 °C and 37 °C compared to the control group.

**Figure 5:**
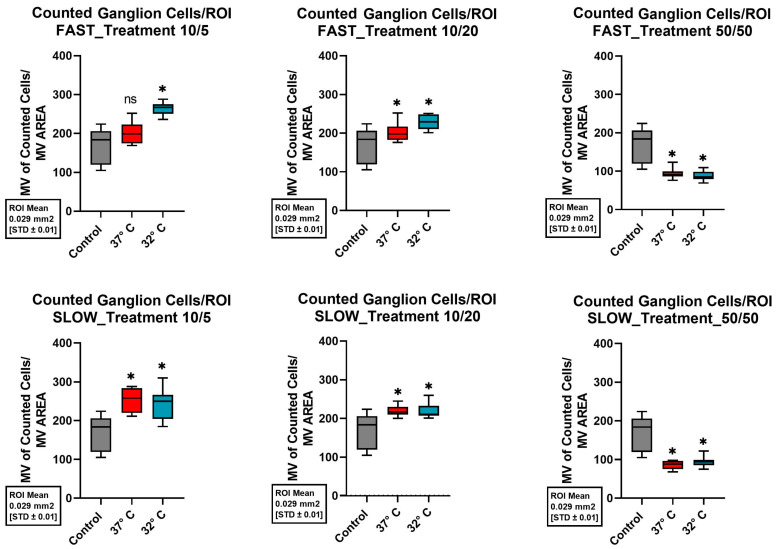
Comparison of mean cell preservation per region of interest (ROI) in the SGNs from different cultures. The columns show the different concentrations of Bdnf and Ntf3 in the culture medium. The top row shows the results for the quickly dissected cochleae, while the bottom row represents the slowly dissected cochleae. In each graph, the control group is shown with a gray boxplot. Cultures at 37 °C are in red, and those at 32 °C are in blue. An asterisk above the boxplot indicates a statistically significant difference compared to the control group. The label “ns” (not significant) above the boxplot indicates that there was no significant difference compared to the control group.

Regardless of the culture temperature and the speed of the cochlea dissection, the addition of neurotrophins at the 50/50 concentration (50 ng/mL Bdnf and 50 ng/mL Ntf3) to the culture medium consistently resulted in a significant decrease in cell survival when compared to the control group (all *p*-values = < 0.001). Figure 5 shows the corresponding boxplots of the cell survival of the different treatments in the SGNs.

### 2.2. Organ of Corti

**Figure 6:**
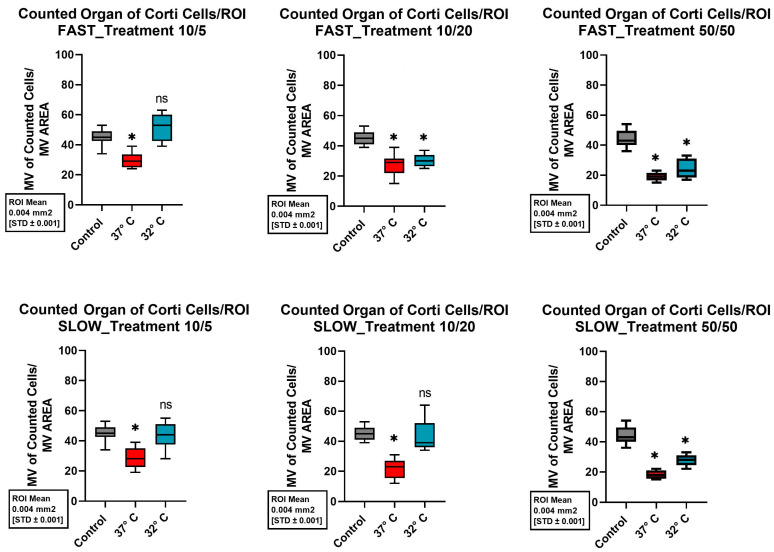
Comparison of mean cell preservation per ROI in the organ of Corti (OC) from different cultures. The columns show the different concentrations of Bdnf and Ntf3 in the culture medium. The top row shows the results for the quickly dissected cochleae, while the bottom row represents the slowly dissected cochleae. In each graph, the control group is shown with a gray boxplot. Cultures at 37 °C are in red, and those at 32 °C are in blue. An asterisk above the boxplot indicates a statistically significant difference compared to the control group. The label “ns” above the boxplot indicates that there was no significant difference compared to the control group.

In the OC, a mean of 45 ± 5.41 (SD) cells per exanimated unit was obtained in the control group (37 °C, no neurotrophins). Only the combination of the quickly dissected cochlea, the addition of 10 ng/mL Bdnf and 5 ng/mL Ntf3, and cultivation at 32 °C resulted in similarly good cell conditions, preserving a mean of 51.89 ± 8.92 (SD) cells per unit. Even when more cells were preserved with the corresponding modifications, there was no statistically significant difference. Figure 6 shows an overview of the total cells obtained in the OC for the different cultures performed. Again, adding 50 ng/mL Bdnf and 50 ng/mL Ntf3 consistently led to a significant drop in cell survival at both temperatures compared to the control group (all *p*-values < 0.001).

**Figure 7:**
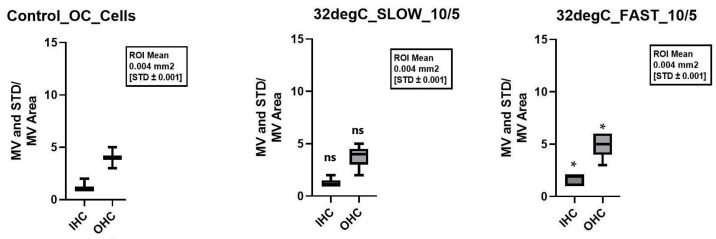
Comparison of the mean cell preservation per region of interest (ROI) in the organ of Corti (OC) under different culture conditions. The figure displays the number of preserved IHCs and OHCs per area. The **left** panel shows the preserved IHCs and OHCs of the control group, while the middle and right panels represent cultures treated with 10 ng/mL Bdnf and 5 ng/mL Ntf3 at 32 °C under slow (**middle**) and fast (**right**) cochlea dissection conditions. Asterisks denote statistically significant differences compared to the control group, while “ns” indicates no significant difference.

Nevertheless, when considering not only the total cells preserved but also the individual cell types, statistically significant better cell preservation of the inner hair cells (IHCs) and outer hair cells (OHCs) was seen when appropriately cultured with 10 ng/mL Bdnf and 5 ng/mL Ntf3 at 32 °C after a fast cochlea dissection. In the control group, 1.11 ± 0.11 IHCs and 4 ± 0.17 OHCs were preserved, while 1.67 ± 0.17 IHC (*p* = 0.015) and 4.89 ± 0.35 OHC (*p* = 0.042) were preserved with the modifications above. Figure 7 presents the graphs of these results.

Quick dissection at 32 °C with neurotrophins for the combination of 10 ng/mL Bdnf plus 20 ng/mL Ntf3 and 50 ng/mL Bdnf plus 50 ng/mL Ntf3 showed similar significance for the preservation of IHCs (*p*-value 0.001 and <0.001). For the OHCs, the significance of a quick dissection was only observed when using neurotrophins at a concentration of 10 ng/mL Bdnf and 20 ng/mL Ntf3 and 32 °C (*p*-value 0.004). Figure 3 shows the better structural preservation of hair cells in immunofluorescence staining. A hair cell marker for Myo7a, which is exclusively found in hair cells, was used.

### 2.3. Stria Vascularis/Spiral Ligament

For the cells of the SV/SL, the control group preserved 252.89 ± 32.54 cells. The addition of 10 ng/mL Bdnf and 5 ng/mL Ntf3 significantly improved cell survival, preserving 311 ± 52.85 cells at 37 °C (*p* = 0.013) and 336.11 ± 38.50 cells at 32 °C (*p* = < 0.001).

**Figure 8:**
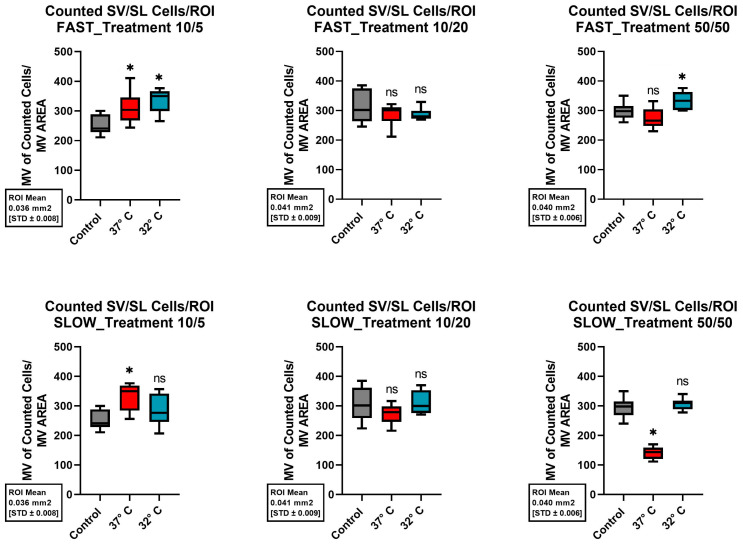
Comparison of the mean cell preservation per ROI in the stria vascularis/spiral ligament (SV/SL) from different cultures. The columns show the different concentrations of Bdnf and Ntf3 in the culture medium. The top row shows the results for quickly dissected cochleae, while the bottom row represents slowly dissected cochleae. In each graph, the control group is shown with a gray boxplot. Cultures at 37 °C are in red, and those at 32 °C are in blue. An asterisk above the boxplot indicates a statistically significant difference compared to the control group. The label “ns” above the boxplot indicates that there was no significant difference compared to the control group.

The addition of Bdnf and Ntf3 at concentrations of 10/20 ng/mL and 50/50 ng/mL did not lead to a significant reduction in cell preservation, as previously described. Figure 8 provides a graphical summary of the changes in cell preservation due to the different culture conditions.

A comparison of the structural preservation in the different areas of the cochlea at different culture conditions showed that the combination of a quick cochlea dissection, adding 10 ng/mL Bdnf and 5 ng/mL Ntf3 to the culture medium, and culturing at 32 °C achieved the best cell survival in all areas of the cochlea. The combination of normothermia and a slow cochlea dissection resulted in the worst structural preservation.

## 3. Discussion

The combination of a quick dissection of the cochlea, hypothermic culture conditions at 32 °C, and the addition of Bdnf and Ntf3 resulted in the best structural preservation.

Rapid removal of the cochlea shows better structural preservation of the inner ear than slow removal, despite a difference of only a few minutes between the methods. The death of the mouse terminates the blood flow toward the cochlea, and the connection to the brain is divided. The resulting hypoxia starts the signal apoptosis cascade and progresses until the cochlea reaches the culture medium [14]. The cellular changes due to hypoxia of the cochlea have been well studied [14,15,16]. Hypoxia leads to a decrease in inner and outer hair cells, with both types being equally sensitive to this condition. In addition to the lack of oxygen, the reduction in glucose has to be mentioned. The combination of glucose deprivation and oxygen deficiency further exacerbates the loss of inner and outer hair cells, with hypoxia being the determining factor [17]. This finding is in agreement with the results of this study. Under the same culture conditions, more hair cells could be preserved with quick dissection. In addition to hair cells, hypoxia initiates a loss of SGNs.

The extent of cellular changes depends on the duration of ischemia, with longer ischemia leading to greater structural loss [14,18]. Consequently, the shortest possible period from explantation, preparation, and transfer to the culturing medium needs to be encouraged. The prompt removal of the cochlea has a positive impact on the microstructures of the inner ear and results in the preservation of the ischemia-sensitive hair cells and neuronal cells. This time is very dependent on the individual skill and routine of the examiner.

In addition to preventing ischemia, neurotrophins may contribute to improved structural preservation. Neurotrophins have been extensively studied for their ability to prevent the degeneration of auditory neurons in deafened animals and to promote the regrowth of nerve fibers after destruction. Various neurotrophic factors, such as Bdnf [19,20,21,22,23,24] and Ntf3 [24,25], have been investigated. In addition to Bdnf and Ntf3, other factors, such as NGF, Neurotrophin 4, Neurotrophin 5, ciliary neurotrophic factor (CNTF), glial cell line-derived neurotrophic factor (GDNF), and members of the fibroblast growth factors (FGF) family, are also known to play a role in the survival regulation of cochlear and vestibular cells [26].

Bdnf and Ntf3 are both naturally expressed in the adult cochlea [27,28]. Both neurotrophins are necessary for the maintenance of cochlea innervation, as the simultaneous loss of both results in greater innervation loss compared to the loss of Bdnf alone [11]. The spatial and temporal expressions of these neurotrophins are critical for cochlear development. As demonstrated by Fariñas et al., Ntf3 is essential for the survival of neurons in the basal cochlear turn, as its absence leads to a complete loss of neurons in this region. Bdnf, on the other hand, is predominantly expressed in hair cells and follows an apical-to-basal expression gradient during development, whereas Ntf3 is primarily found in supporting cells [29]. It is important to note that TrkB and TrkC, the receptors for Bdnf and Ntf3, respectively, are not only required for neurotrophin signaling but are also independently essential for SGN development. Mice with incomplete deletions of both TrkB and TrkC exhibit cochlear phenotypes similar to those observed in Bdnf and Ntf3 knockouts, highlighting the critical nature of these receptor-ligand systems [13,30]. Thus, the preservation of SGNs in culture may reflect not only the presence of neurotrophins but also the intact signaling through these Trk receptors.

In the present study, only the combined influence of Bdnf and Ntf3 was tested. However, the distinct roles of these neurotrophins in different cochlear regions suggest that targeted neurotrophic support may be necessary to optimize neuronal survival and connectivity. Vink et al. described the combination of Bdnf and Ntf3 to be superior in SGNs preservation compared to treatment with either Bdnf or Ntf3 alone [31]. They also achieved better cell preservation of the SGNs with Bdnf therapy alone versus Ntf3 therapy alone [31].

The present study, which evaluated the previously established whole cochlea organ culture procedure [3], found that the addition of neurotrophic factors Bdnf and Ntf3 together improved histopathological cell preservation. However, the dose of neurotrophins in the culture media is crucial, as excessively high neurotrophin concentrations appeared to negatively affect the culture outcome. Significantly fewer structures were preserved with the addition of 50 ng/mL Bdnf and 50 ng/mL Ntf3 compared to cultures without neurotrophins. Although a negative effect due to overexpression of Ntf3 has been observed in previous studies [32,33], there are insufficient data to confirm that an excess of Bdnf negatively impacts cochlear cell survival. Since worse cell survival in the OC and the SGNs was observed in this study when using 50 ng/mL Bdnf and 50 ng/mL Ntf3, we hypothesize that this effect may be due to excessive Ntf3. Nevertheless, this remains unclear, as the neurotrophins were only tested together and not separately. However, while the high concentration of 50 ng/mL Bdnf and 50 ng/mL Ntf3 resulted in reduced cell survival in the OC and SGNs, it improved cell survival in the SV. This discrepancy may be related to differences in the cellular response to neurotrophins in distinct cochlear structures. Future studies should investigate these effects further by examining the individual impact of Bdnf and Ntf3 on different cochlear regions. Additionally, the SV could be examined in greater detail. Specifically, the addition of pendrin may help maintain the endocochlear potential, potentially leading to better structural survival [4]. Future studies should consider this approach to improve the analysis and understanding of the SV under similar conditions.

Mild hypothermia was investigated as a third factor to potentially improve culture. Previous studies have connected mild hypothermia to a reduction in the inflammatory immune response and a decrease in intracellular apoptosis. This mechanism could be linked to hearing preservation and improved hair cell survival after various ear traumas [8,34,35,36,37,38].

The best protective effect of mild hypothermia during cultivation was achieved in combination with a fast cochlea dissection and the addition of neurotrophins.

Considered individually, a rapid dissection of the cochlea, the addition of neurotrophins, and mild hypothermia each showed a positive effect on the structural preservation of the inner ear organs. However, the combination of all three factors combined for a better structural preservation of all the different cell types of the inner ear in a 10-day-old C57BL6/J model. Therefore, this combination of protective factors seems to be crucial. Since the combination of various positive factors with various control factors resulted in inconclusive cell survival, as shown in Figure 5, Figure 6, Figure 7 and Figure 8, it is not possible to say which one of these factors had a greater effect. Therefore, an equivalence of the tested protective factors needs to be assumed.

The preparation time of the cochlea, the culture temperature, and the addition of neurotrophins to the culture medium showed only a small effect on the organ culture of the cochlea when considered individually. The combination of these factors, however, represents a strong positive influence.

## 4. Materials and Methods

The study protocol was designed to optimize the previously described whole-organ cochlea culture protocol [3] regarding structure preservation and culturing age. It was aimed to optimize the culture conditions for 10-day-old C57/BL6J mice. After explantation, the cochleae were transferred into a culture medium. The cochleae were divided into fast and slow preparation groups. The fast group represented the first explanted cochlea, and the slow group represented the second. A total of four different culture media were compared, consisting of different concentrations of neurotrophins (mouse-derived proteins). The exact concentrations are shown in Table 1.

Finally, the organs were cultured as described below at either 37 °C or 32 °C. After a 24 h culturing period, the cochleae were processed and stained using hematoxylin–eosin (HE) and toluidine blue staining. In addition, immunostaining for Myo7a, phalloidin, and ß-III-tubulin was performed.

The structure preservation was determined in three different areas and interpreted in a blinded manner by two independent specialists (SJ, ASF). The areas were OC, SGNs, and SV/SL. For each experiment performed, three cochleae were studied.

### 4.1. Animals

For this study, C57/BL6J mice were obtained from the Charles River Company in Freiburg, Germany. The mice were maintained at the Innsbruck animal facility with unlimited access to food and water. Housing, feeding, breeding, and handling of the mice were carried out according to federal/institutional guidelines, with the approval of the local government. Mice of each sex were used for this study. The dissection of the inner ear organs took place on postnatal day 10. The animals were anesthetized through intraperitoneal injections of ketamine hydrochloride (Graeub^®^, Senden-Bösensell, Germany) (67.5 mg/kg body weight), xylazine hydrochloride (Bayer^®^, Leverkusen, Germany) (5.4 mg/kg body weight), and atropine sulfate (Nycomed^®^, Linz, Austria) (0.085 mg/kg), and then euthanized by rapid cervical dislocation.

### 4.2. Dissection of the Inner Ear Organs

The dissection was performed under sterile conditions. Juvenile mice (postnatal day 10) were deeply anesthetized and sacrificed by decapitation. Starting at the foramen magnum, the skull was cut along the sagittal plane, followed by the removal of the brain. Then, the bone plates surrounding the cochlea were removed so that the cochlea and vestibular organ could be harvested. Subsequently, the oval and the round window were opened. Then, the bone coverage of the apex was broken along the basal and middle turn of the cochlea. Finally, the bony shell of the scala tympani was opened and removed, resulting in a 180° opening along the basal turn.

After the dissection, the inner ear organs were finally placed on a fresh Petri dish and carefully rinsed with neurobasal medium (Gibco^TM^, ThermoFisher Scientific^®^, Schwerte, Germany, cat-nr. 21103049). Depending on the duration of preparation—fast or slow—the cochleae were cultured separately. The cochleae in the fast group were transferred to the culture medium approximately 5 min after decapitation, whereas those in the slow group were placed in the culture medium approximately 10 min after decapitation. Therefore, there was an average of 5 min between the quickly and slowly harvested cochlea.

### 4.3. Culture Medium

Preparation of the culturing medium was performed the day before the harvest of the inner ears. The base liquid used for the culturing medium was neurobasal medium, to which 5 mM L-Glutamine, 10 mM HEPES, 100 U/mL Penicillin G, and B27 supplement (Gibco^TM^, Thermo Fisher Scientific^®^, Schwerte, Germany) were added. Subsequently, the pH was adjusted to 7.4 by adding 1 M NaOH. Then, different concentrations of NT-3 (PeproTech^®^ EC, Ltd., London, UK) and Bdnf (PeproTech^®^ EC, Ltd., London, UK) were added and tested, as described below.

Four different culture mediums were included in this study, as listed in Table 1.

### 4.4. Culturing of the Dissected Cochlea

Culturing was performed at 37 °C or 32 °C in a humidified 5% CO_2_/95% water-jacketed incubator (Scientific^TM^, Thermo Fisher Scientific^®^, Marietta, OH, USA), which provided stable temperature gradients. After the dissection of the inner ear organs, they were placed in a 10 mL disposable rotary vessel filled with warmed neurobasal medium. Then, the vessels were mounted to the rotating bioreactor machine, which rotated them clockwise at 50–52 rounds per minute (rpm) to simulate zero gravity. Depending on the size and weight of the cochlea, different rounds per minute were used to ensure that the cochlea did not collide with the wall of the vessel while rotating. Culturing was performed for 24 h.

### 4.5. Fixation, Decalcification, and Freezing

After incubation, the cochleae were transferred to 2% (weight/volume) paraformaldehyde (PFA), which was further diluted with neurobasal medium to a pH of 7.4. Subsequently, the cochleae were decalcified using 10% ethylenediaminetetraacetic acid (EDTA) and phosphate-buffered saline (PBS 1×) at a pH of 7.4 at 37 °C. The cochleae were placed in this mixture for four hours. Before and after the process of decalcification, the cochleae were washed three times in PBS 1×. Then, the cochleae were frozen using a cryoprotected freezing method [39]. For this purpose, the inner ears were rinsed with PBS 1× and then shaken with 10% sucrose and 15% sucrose (dissolved in PBS 1×) at room temperature. Subsequently, the inner ears were placed in a mixture consisting of half 15% sucrose dissolved in PBS 1× and half OCT and shaken. They were frozen in a mixture of CO_2_ and EtOH. This was followed by cryocutting, producing 5–7 μm thick sections.

### 4.6. Staining

According to standard protocols, HE staining was performed. In addition, the cultured cochleae were immunostained using the Roche Ventana Discovery System (Roche Diagnostics^®^, Rotkreuz, Switzerland). The markers used were phalloidin FITC (Sigma-Aldrich^®^, Vienna, Austria, cat. No. P5282) to stain F-actin in supporting cells, anti-Myo7a (Proteus Biosciences^®^ Inc., Ramona, CA, USA, cat. No. 25-6790) to stain hair cells, which indicates the condition of inner hair cells (IHCs) and outer hair cells (OHCs), and anti-ß-III-tubulin (Abcam^®^ plc., Cambridge, UK, cat. No. ab52623) to stain nerve fibers and ganglia cells. Concentrations of 1:100 for the ß-III-tubulin antibody, 1:100 for the Myo7a antibody, and 1:20 for the phalloidin FITC were applied. Alexa Rabbit 594 (Life Technologies^TM^, Invitrogen^®^ Inc., Darmstadt, Germany, ref. A21207, lot. 2313074) was used as a secondary antibody at a concentration of 1:200.

The exact stain immunohistochemistry procedures have been described in previous publications [27,40].

### 4.7. Quantitative and Statistical Evaluation

To quantify the structural preservation of the cochlea, histological sections were investigated in detail with the help of TissueQuest (version 7.0.1.139, Tissuegnostics Ltd., Vienna, Austria). We used DAPI to stain the cell nuclei (Life Technologies^TM^, Invitrogen^®^, Inc., Darmstadt, Germany) on the master canal, Myo7a as a marker for hair cell survival, and ß-III-tubulin as a marker for ganglion cell survival. TissueQuest then automatically detected the differently stained cells. We manually dissected the sample into three different regions of interest (ROIs), which were the OC, SGNs, and SV/SL. The number of cells per region was calculated in TissueQuest and also counted manually. Each count was based on three replicates with three repetitions.

For statistical evaluation, the mean values and standard deviations were calculated using Graphpad Prism (version 9.4.1681 by Graphpad Software Inc., San Diego, CA, USA) and “Statistical Package for the Social Sciences” version 26.0.0.0 from IBM (SPSS Statistics; IBM, Armonk, NY, USA). Subsequently, an unpaired *t*-test was used to calculate possible significances between the different treatments. A significance level of α = 0.05 was set for the statistical calculations.

## Figures and Tables

**Table 1:** The different concentrations of neurotrophins in the culture media.

Added Neurotrophin	Control	10/5	10/20	50/50
Bdnf	none	10 ng/mL	10 ng/mL	50 ng/mL
Ntf3	none	5 ng/mL	20 ng/mL	50 ng/mL

## Data Availability

The raw data supporting the conclusions of this article will be made available by the authors upon request.

## Abbreviations

The following abbreviations are used in this manuscript:

Bdnf	Brain-derived neurotrophic factor
HE	Hematoxylin–eosin
NA	Numerical Aperture
Ntf3	Neurotrophin 3
OC	Organ of Corti
PBS 1×	Phosphate-buffered saline
SD	Standard deviation
SL	Spiral ligament
SGNs	Spiral ganglion neurons
SV	Stria vascularis
